# Bridging the Gap: Using Implementation Science to Support Communication in Dementia Care

**DOI:** 10.1093/geroni/igaf122.172

**Published:** 2025-12-31

**Authors:** Natalie Douglas, Allison Lindauer

## Abstract

Effective dementia care relies on person-centered, evidence-based approaches that improve outcomes for people living with dementia and their care partners. A key challenge in implementing dementia care strategies is that it relies on effective communication—yet communication impairments are a core feature of dementia. Without addressing these impairments, even well-designed interventions may be difficult to sustain, adapt, or scale. Despite the critical role of communication in dementia care, speech-language pathologists (SLPs) remain underutilized in implementation efforts. This symposium explores how communication-based strategies can be successfully integrated into dementia care using implementation science principles, with lessons for researchers, clinicians, and policymakers working to enhance care delivery. First, a case study illustrates how unaddressed communication challenges create barriers to dementia care implementation. The second study maps care partner-reported distressing behaviors to speech-language pathology interventions, highlighting how communication-based strategies can be tailored to both individual and contextual needs. The third study presents a scalable, technology-based intervention designed to foster social engagement for dementia care partners, illustrating the importance of addressing communication needs beyond direct patient care. The fourth study evaluates a real-world adaptation of Dementia Collaborative Coaching, where care providers are trained in communication strategies to improve dementia care delivery in resource-limited settings. Together, these studies illustrate how applying implementation science principles to communication-based dementia care strategies can enhance care outcomes, improve intervention sustainability, and reduce care partner burden. These findings have broad implications for gerontologists, healthcare professionals, and policymakers committed to improving dementia care.

